# A missed diagnosis of sputum crust with fiberoptic bronchoscope causing extubation failure: a case report

**DOI:** 10.1186/s12890-023-02457-w

**Published:** 2023-05-02

**Authors:** Kun Qian, Yiyong Wei, Xingkui Liu, Zhengfu Li, Song Cao, Dan Wen, Junhua Shi, Yu Zhang, Yinan Zhang

**Affiliations:** 1 Department of Anesthesiology, Affiliated Hospital of Zunyi Medical University, 149 Dalian Street, Guizhou 563000 Zunyi, China; 2 College of Anesthesiology, Zunyi Medical University, Guizhou 563000 Zunyi, China; 3 Respiratory Department, Affiliated Hospital of Zunyi Medical University, Guizhou 563000 Zunyi, China; 4 Department of Pain Medicine, The Affiliated Hospital of Zunyi Medical University, Guizhou 563000 Zunyi, China; 5 Department of Radiology, Affiliated Hospital of Zunyi Medical University, Guizhou 563000 Zunyi, China

**Keywords:** Fiberoptic bronchoscope, Sputum crust, Extubation failure, Postoperative pulmonary complications, High-resolution chest computed tomography

## Abstract

**Background:**

Fiberoptic bronchoscopy (FOB) and bronchoscopic biopsy are the established methods for diagnosing and treating sputum crust. However, sputum crust in concealed locations can sometimes be missed or undiagnosed, even with bronchoscopy.

**Case presentation:**

We present the case of a 44-year-old female patient who experienced initial extubation failure and postoperative pulmonary complications (PPCs) due to the missed diagnosis of sputum crust by FOB and low-resolution bedside chest X-ray. The FOB examination showed no apparent abnormalities prior to the first extubation, and the patient underwent tracheal extubation 2 h after aortic valve replacement (AVR). However, she was reintubated 13 h after the first extubation due to a persistent irritating cough and severe hypoxemia, and a bedside chest radiograph revealed pneumonia and atelectasis. Upon performing a repeat FOB examination prior to the second extubation, we serendipitously discovered the presence of sputum crust at the end of the endotracheal tube. Subsequently, we found that the sputum crust was mainly located on the tracheal wall between the subglottis and the end of the endotracheal tube during the “Tracheobronchial Sputum Crust Removal” procedure, and most of the crust was obscured by the retained endotracheal tube. The patient was discharged on the 20th day following therapeutic FOB.

**Conclusion:**

FOB examination may miss specific areas in endotracheal intubation (ETI) patients, particularly the tracheal wall between the subglottis and distal end of the tracheal catheter, where sputum crust can be concealed. When diagnostic examinations with FOB are inconclusive, high-resolution chest CT can be helpful in identifying hidden sputum crust.

## Background

Sputum crust refers to the presence of dry, granular, or lumpy sputum, which is a common occurrence in the airways during the perioperative period for patients undergoing cardiac surgery. Despite significant improvements in patient care, sputum crust remains linked to several adverse outcomes, including increased mortality risk, postoperative pulmonary complications (PPCs), a higher incidence of extubation failure, longer stays in the cardiac intensive care unit (CICU), and prolonged mechanical ventilation [1]. In some cases, sputum crust can lead to the formation of a membranous tracheal solidified-sputum flap that functions as a “one-way valve,“ obstructing the airway and causing fatal consequences. Managing patients with sputum crust requires significant healthcare resources and multidisciplinary care, involving anesthesia, surgery, respiratory medicine, and CICU [2].

Prompt diagnosis of sputum crust is crucial given the associated risks and resource demands. While fiberoptic bronchoscopy (FOB) plays a central role in diagnosis, it may miss sputum crust located in concealed areas such as the blind area between the subglottis and the tip of the tracheal tube in endotracheal intubation (ETI) patients [3]. Sole reliance on FOB may result in missed or delayed diagnosis, and imaging examinations such as high-resolution contiguous chest CT should also be considered. In patients with suspected sputum crust, high-resolution CT is a valuable diagnostic tool, offering rapid and accurate diagnosis [4].

## Case presentation

A 44-year-old female patient presented with a history of palpitations for over a year following a cold. Echocardiography revealed severe aortic valve regurgitation and left ventricular systolic dysfunction. An ECG showed left ventricular hypertrophy and strain. A routine chest X-ray was performed, and the preoperative diagnoses were aortic valve insufficiency and Takayasu’s arteritis (TAK). The patient underwent AVR with cardiopulmonary bypass (CPB) under general anesthesia with endotracheal intubation (ETI) on April 25, 2021. The surgery lasted 285 min, with a CPB time of 110 min and an aortic cross-clamp time of 74 min. The procedure was completed with relatively stable vital signs, and the patient with ETI was transferred to the CICU after surgery.

After successfully implementing tracheal extubation strategies, the patient’s endotracheal tube was removed two hours following AVR. These strategies included routine fiberoptic bronchoscopy, closed endotracheal suction, and successful weaning criteria and spontaneous breathing trial (SBT). The patient experienced an irritating cough and mild wheezing immediately after the first extubation, but without dyspnea or cyanosis. Arterial blood gas examination revealed a pH of 7.43, PaCO_2_ of 48 mmHg, and PaO_2_ of 86 mmHg. However, on postoperative day (POD) 1, the patient developed persistent coughing and severe hypoxemia. The patient underwent orotracheal intubation using a video laryngoscope, but resistance was encountered after the tracheal tube cuff passed through the glottis. Instant parameters after reintubation revealed SpO_2_ of 90%, pH of 7.48, PaCO_2_ of 53 mmHg, and PaO_2_ of 65 mmHg. On POD 2, the patient received invasive ventilatory support with supplemental sedation and analgesia, and SpO_2_ was monitored at 98–100% (FiO_2_ rates of 30–50%). Due to poor cardiac function, a bedside chest X-ray was performed instead of a chest CT. The X-ray revealed post-cardiac surgical changes, cardiac enlargement, pneumonia, left pleural effusion after closed thoracic drainage, left lower lung lobe partial transudation, and fibrosis with atelectasis.

On POD 3, we planned a second extubation and used FOB to assess the respiratory tract during the peri-extubation period to prevent extubation failure. During this examination, we incidentally discovered a crust-like sputum protrusion firmly attached to the tip of the endotracheal tube, which could not be suctioned out. Therefore, we scheduled the patient for “Tracheobronchial Sputum Crust Removal”.

**Fig. 1:**
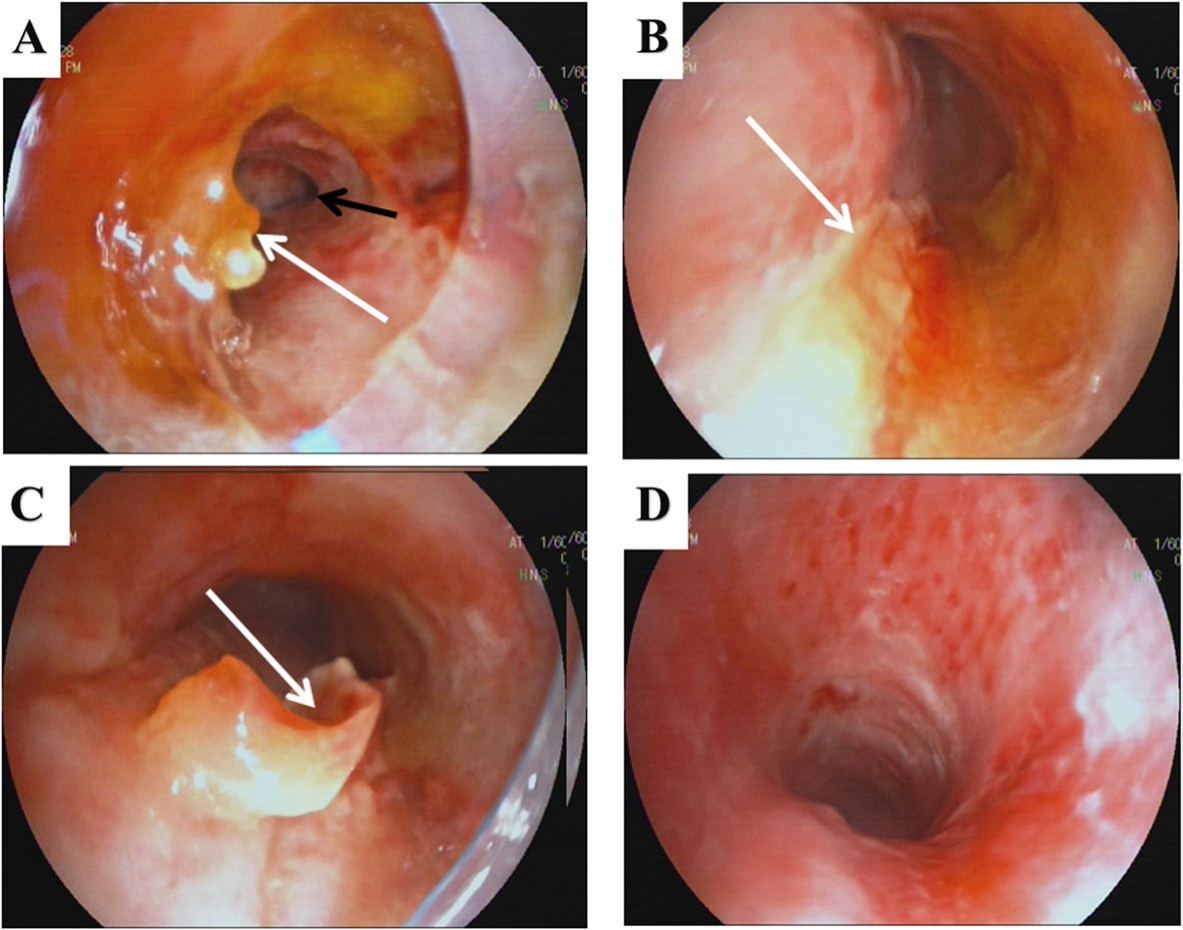
Bronchoscopic images taken before and after therapeutic FOB. In panels **A**, **B**, and **C**, white arrows indicate the presence of sputum crust, while the black arrow points to the right bronchus. Panel **D** illustrates that the tracheal cartilages become visible

After the discovery of the sputum crust-like protrusion attached to the endotracheal tube, a respiratory physician used FOB to examine the patient’s respiratory tract. The tracheal catheter was replaced with a laryngeal mask intraoperatively. FOB revealed that the sputum crust was almost entirely covered by the tracheal catheter and extended from the subglottic area 2.0 cm towards the superior 2.5 cm of the carina, occupying approximately 50% of the normal tracheal lumen (Fig. 1A and B). Flexible foreign body forceps were used under FOB guidance to remove the large obstructing crusts (Fig. 1C), after which the lumen was unobstructed (Fig. 1D). The laryngeal mask was replaced by an endotracheal tube under FOB guidance, and the patient was transferred to the CICU for further treatment. Intraoperative vital signs were stable, and the total operation time was 25 min. Pathological examination of the crust showed acute inflammatory secretions, primarily fibrin-like exudates.

**Fig. 2:**
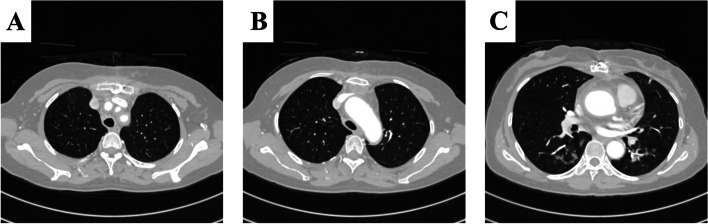
High-resolution chest CT images showing primary outcomes following therapeutic fiberoptic bronchoscopy. Panels **A**-**C** display trachea, bronchus, and basal bronchus, respectively (indicated by red arrows), while the great vessels are highlighted by the yellow arrow

**Fig. 3:**
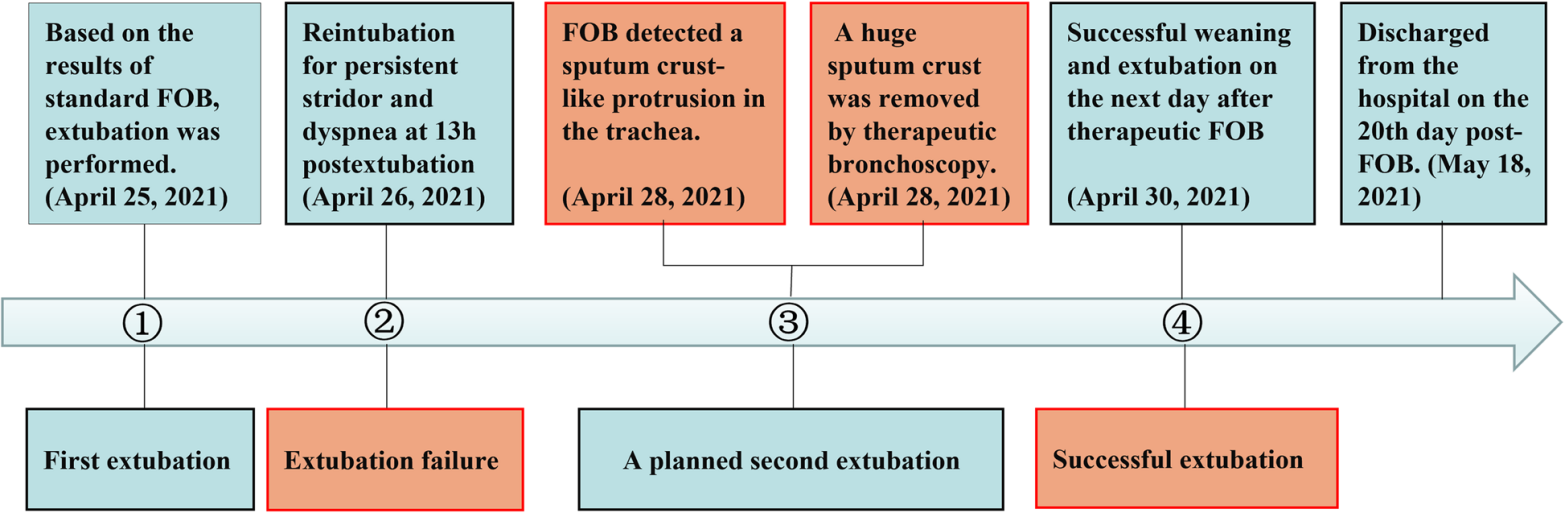
Timeline illustrating the diagnosis and intervention process for a large, circular attached sputum crust

She was extubated on April 30, 2021, the second day after the “Tracheobronchial Sputum Crust Removal” procedure. On the 14th day, a high-resolution thin-Sect. (0.8 mm) chest CT was performed and showed that there was little transudation in the bilateral lower lungs compared to the chest radiograph findings on April 26, 2021. The CT images demonstrated that the sputum crust had completely disappeared, with unobstructed bronchus and smooth wall, and left lower lobe recruitment maneuvers were observed (Fig. 2). The patient’s condition improved, and she was discharged on the 28th day after admission, as shown in Fig. 3. Overall, the patient had a favorable outcome.

## Discussion and conclusion

Sputum crust is a common complication in patients receiving mechanical ventilation via endotracheal intubation (ETI). It can lead to airway obstruction, pneumonia, atelectasis, respiratory failure, extubation failure, and even increased mortality. Large sputum crusts may completely obstruct the tracheal tube or lumen, causing life-threatening asphyxia and cardiopulmonary arrest. Tracheal rupture after sputum scab formation may also increase mortality, according to a study by Capasso and colleagues [5].

Since missed or misdiagnosed sputum scabs can result in serious adverse events, accurate diagnosis and prompt treatment are crucial for improving prognosis. Clinical presentations of sputum crusts are non-specific and generally include symptoms such as irritating cough/bucking, high airway pressure, wheezing/stridor, dyspnea or hypoxemia, pulmonary atelectasis, and pneumonia. Invasive FOB examination is the gold standard for diagnosis [3]. However, the distance from the subglottis to the tip of the endotracheal tube is a blind area for FOB examination in ETI patients, making early recognition of sputum scabs even more challenging.

In this case, the initial sputum crust was concealed and completely covered by the tracheal tube, which was not detected during the first bronchoscopy. Additionally, a lower-resolution bedside chest X-ray was performed instead of a thin-layer chest CT due to cardiac dysfunction after AVR. Compared with chest radiography, chest CT has a higher resolution for phlegm scabs [4]. Moreover, the patient’s clinical manifestations after extubation were atypical, despite mild wheezing and irritating cough. A colossal sputum crust is associated with a relatively long mechanical ventilation time and generally causes increased airway resistance or complete obstruction. However, these prominent features did not occur in our case.

To sum up, this patient’s missed diagnosis and delayed treatment can be attributed to three factors: the concealed location of the sputum crust, the use of a lower-resolution bedside chest X-ray, and atypical clinical manifestations. Early recognition and prompt treatment of sputum crusts are essential for improving prognosis, and chest CT should be considered for patients suspected of having sputum crusts.

Advanced diagnostic tools have been applied to improve the accuracy of sputum scab diagnosis, with high-resolution CT being one of the most beneficial methods [4]. Traditionally, sputum crust diagnosis relied on relatively specific symptoms, X-rays, or even diagnostic thoracotomy. However, recent studies have shown that FOB and high-resolution chest CT are superior in achieving an accurate diagnosis [3, 4]. FOB has a high diagnostic accuracy but is an invasive procedure and can miss undetectable areas [3]. In contrast, high-resolution thoracic CT is a non-invasive technology that provides a primary diagnosis [4]. The CT scan can show sputum crusts arranged in branching structures resembling the branches of a tree or in granular or irregular strip-like shapes. In our case report, FOB examination revealed no apparent abnormalities before the first extubation, and the patient did not undergo chest CT due to her poor cardiac function after the first extubation failure, resulting in a missed diagnosis of sputum crust. Although sputum crusts are common in clinical practice, few articles have reported on their diagnosis using high-resolution CT, which may provide a new direction for future research. Therefore, in patients with suspected hidden sputum scabs, combining high-resolution chest CT with FOB diagnostic examinations when the latter are inconclusive can be helpful.

In our case, airway failure was identified as a contributing factor to sputum scab formation, which was mainly caused by several factors. Firstly, inspired non-humidified gas led to the solidification of sputum in the tracheobronchial tree. Secondly, cardiac insufficiency caused excessive respiratory secretions, while the insufficient ability of secretions clearance or ineffective cough further exacerbated the condition.

To address the possible pathogenesis of sputum scab formation, we have implemented complementary treatment and prevention strategies. Firstly, we followed the treatment approach reported by Choi and colleagues and utilized FOB to remove sputum solidified casts and performed BAL [6]. Secondly, we emphasized the importance of meticulous airway management techniques for patients at risk of developing sputum scabs. In recent years, the treatment and prevention of sputum crust have focused on measures such as airway humidification, adequate airway suctioning, minimized lung injury (closed circuits during suctioning, non-invasive ventilation after extubation), and other symptomatic treatments (anti-inflammatory, closed thoracic drainage, and improved cardiac function) [7].

In summary, we present a unique case of an endotracheal intubation (ETI) patient with a large sputum crust located between the subglottic area and the end of the endotracheal tube. This condition went undiagnosed by flexible bronchoscopy (FOB), which resulted in serious adverse events. From this case, readers can gain insight into the specific limitations of FOB examinations in ETI patients. Moreover, this study offers a new approach for improving diagnostic accuracy by incorporating high-resolution chest CT scans for this patient population.

## Data Availability

The datasets used and analyzed during the current study are available from the corresponding author upon reasonable request.

## Abbreviations

AVR: Aortic valve replacement
ETI: Endotracheal intubation
CICU: Cardiac intensive care unit
CT: Computed tomography
FOB: Fiberoptic bronchoscopy
PPCs: Postoperative pulmonary complications
POD: Postoperative day
SBT: Spontaneous breathing trial
TAK: Takayasu's arteritis

## References

[1] Burns KEA, Rizvi L, Cook DJ (2021). Ventilator Weaning and Discontinuation Practices for critically ill patients. JAMA.

[2] Mathis MR, Duggal NM, Likosky DS, Haft JW, Douville NJ, Vaughn MT (2019). Intraoperative mechanical ventilation and postoperative pulmonary complications after cardiac surgery. Anesthesiology.

[3] Criner GJ, Eberhardt R, Fernandez-Bussy S, Gompelmann D, Maldonado F, Patel N (2020). Interventional Bronchoscopy. Am J Respir Crit Care Med.

[4] Raju S, Ghosh S, Mehta AC (2017). Chest CT signs in Pulmonary Disease: a Pictorial Review. Chest.

[5] Capasso R, Carbone M, Rossi E, Mamone R, Zeccolini R, Reginelli A (2016). A 4-year-old child presenting morning onset of spontaneous tracheal rupture due to bronchial mucous plug occlusion during the nighttime sleep: a case report. J Med Case Rep.

[6] Choi EK, Lee S, Lee D, Park SJ (2018). Successful removal of an intractable mucoid impaction in the bronchus using a Fogarty catheter with flexible bronchoscopy. Saudi J Anaesth.

[7] Dexter AM, Scott JB (2019). Airway Management and Ventilator-Associated events. Respir Care.

